# Left Ventricular Dissecting Hematoma Caused by Tissue Stabilizer

**DOI:** 10.21470/1678-9741-2019-0024

**Published:** 2020

**Authors:** Ashwini Kumar Pasarad, Madhusudhan Madihalli Gopivallabha, Akshay Kumar Singh, Kishore Kolkebaile Sadanand

**Affiliations:** 1 Sagar Hospitals Banashankari, Bengaluru, Karnataka, India.

**Keywords:** Coronary Artery Bypass, Off-Pump Coronary Artery Bypass, Hematoma, Heart Ventricles

## Abstract

Coronary artery bypass grafting is a commonly performed procedure for coronary revascularization. We describe the successful management of left ventricular dissecting hematoma, caused by the tissue stabilizer, while performing off-pump coronary artery bypass graft procedure.

**Table:** 

Abbreviations, acronyms & symbols	
CABG	= Coronary artery bypass grafting
CPB	= Cardiopulmonary bypass
ECG	= Electrocardiogram
EF	= Ejection fraction
LAD	= Left anterior descending artery
LV	= Left ventricle
MI	= Myocardial infarction
OPCAB	= Off-pump coronary artery bypass grafting
RSVG	= Reverse saphenous vein graft
RWMA	= Regional wall motion abnormality
ST	= Segment ECG

## INTRODUCTION

Coronary artery bypass grafting (CABG) is traditionally performed on cardiopulmonary-bypass (CPB). Recently, off-pump coronary artery bypass grafting (OPCAB) has found many takers and many cardiac surgeons have been practising it. Tissue stabilizers have become a vital part of OPCAB. In this study, a rare case of large Left Ventricular (LV) dissecting hematoma caused by Octopus stabilizer is presented, which was managed successfully. A thorough literature search led us to a case of LV dissecting hematoma reported in 2002, but the efforts to manage the case were unsuccessful^[1]^.

### Case Report

A case of a seventy-two-year-old male patient is reported, diagnosed with coronary artery disease and referred for CABG. Coronary-angiogram revealed triple vessel disease. Echocardiogram showed no regional wall motion abnormality (RWMA) and Ejection-fraction (EF) of 55%.

LIMA--Mid-LAD (Left Internal mammary artery-to-mid Left anterior descending artery), RSVG--Distal-LAD (Reverse-saphenous vein-graft-to-Distal Left anterior descending artery) and RSVG-Ramus anastamoses were completed off-CPB, using Octopus(Maquet-Acrobat-i. OM-10000) tissue-stabilizer with 200 mmHg negative-pressure. The other coronaries were diffusely diseased, non-graftable.

Post-grafting, electrocardiogram (ECG) was unremarkable, hemostasis was done. Sternal wiring was done. ST-segment elevation was noted in the lateral-leads on ECG, associated with hemodynamic deterioration. Sternal-wires were cut and chest was re-opened immediately. A large hematoma was noted near the Ramus-graft. CPB was established emergently. Examination revealed the hematoma, measuring about 5X6cm, present at the site of application of the octopus stabilizer during the RSVG-Ramus anastamosis, covering the part of the LV between LAD and Ramus.

A nick was made in the epicardium to evacuate the hematoma and limit the dissection. Rapid re-accumulation of blood was noted underneath the epicardium. Repeated attempts were made to take plication-sutures over the epicaradium but were unsuccessful.

**Fig. 1:**
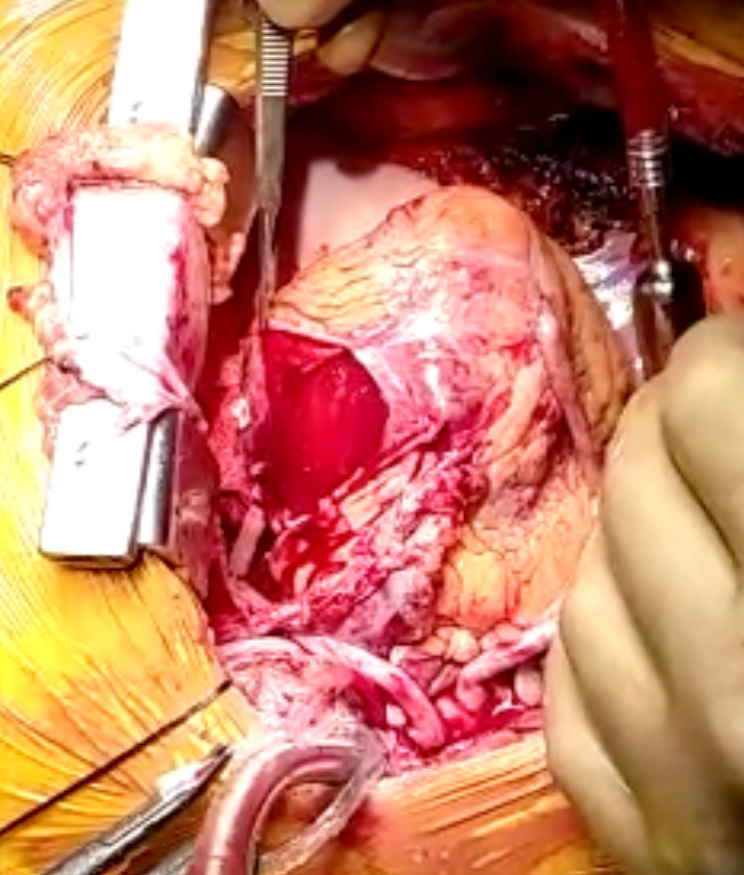
Bare area of myocardium at the site of dissection.

The epicardial-membrane was opened which revealed a bare-area of the myocardium (Figure 1), with multiple bleeders, at the sites of thebesian-vessels. Heart was arrested with cardioplegia.

**Fig. 2:**
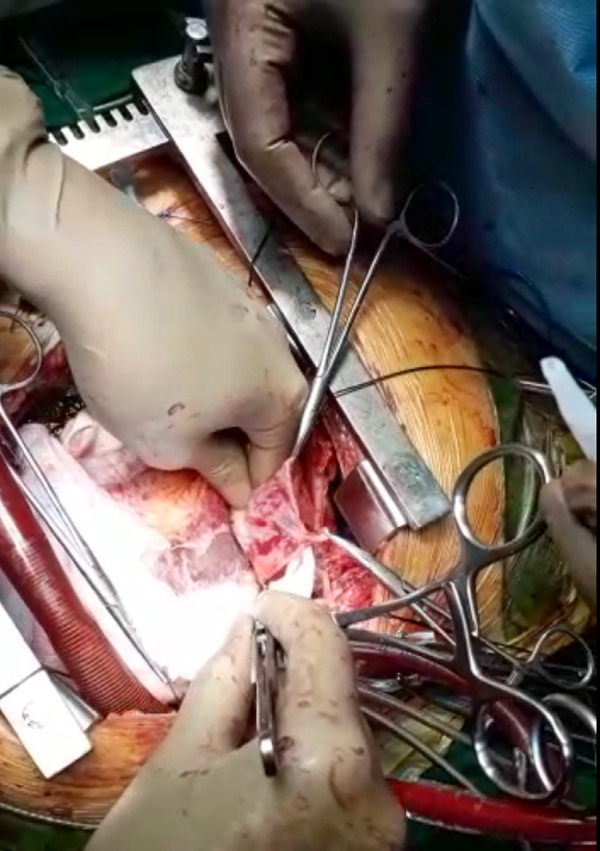
Pericardial patch used to achieve hemostasis using cyanoacrylate.

Once arrested, attempts were made to seal the bleeding-areas with Fibrin-sealant but were unsuccessful. A large autologous-pericardial free-patch was prepared and the myocardium was covered (Figure 2), using Cyanoacrylate-glue. Sufficient contact-time was given. Aortic cross-clamp was removed. Patient was weaned off CPB with intra-aortic balloon-pump. Post-operative echocardiogram revealed minimal pericardial-effusion, RWMA of antero-lateral walls, EF of 45%. Patient was discharged on ninth postoperative day, and examined a week later. Follow-up echocardiogram showed an EF of 50% and RWMA had reduced. The patient continues to be followed up.

## DISCUSSION

After cardiogenic shock, myocardial rupture is the leading cause of in-hospital mortality post-MI^[2]^. LV dissection hematomas, post-CABG, large enough to cause hemodynamic changes, and their successful management have been rarely described. Tissue stabilizers are an integral part of OPCAB and are being used extensively. Octopus stabilizers work on the principle of stabilizing the ventricular wall by applying suction. An important drawback of such suction devices is, sub-epicardial hematoma formation, at the site where suction is applied. Usually these hematomas are small, don’t bleed and resolve on their own, without causing hemodynamic changes^[1]^. We encountered a large expanding LV dissecting hematoma which was managed on CPB. Bleeding was severe and to achieve hemostasis was a challenging task. Hemostasis has been traditionally achieved with suturing, clipping and electrocoagulation. Surgical adhesives are being increasingly used in cardiac surgery. The different types of adhesives include aldehyde-based glues (BioGlue), fibrin-sealants (Tisseel), collagen-based adhesives and Cyanoacrylates^[3]^. Cyanoacrylate-derivates have been successfully used in cardiovascular surgery. Robicsek and colleagues reported using cyanoacrylate to control hemorrhage in critical situations^[4]^. Studies have shown that cyanoacrylate lacks bacterial contamination^[5]^ and has bacteriostatic-activity against most microorganisms^[4]^. Cyanoacrylate has the strongest adhesive power but has the disadvantage of becoming stiff when applied. Hence its use is indicated in desperate situations that cannot be controlled by standard techniques. Cyanoacrylate-glue is one of the cheapest adhesives. It may be seen as an adjunct in cardiac surgery, considering its lifesaving results and documented safety.

Advantages of adhesives include reduced blood loss, hence, the need for blood/products transfusion, reduced periods of CPB/circulatory arrest, total operation time and reduced reoperation rate.

Some documented disadvantages include toxicity, such as tissue-inflammation, immunologic response causing allergy, hypersensitivity reaction or anaphylaxis. Some glues cause adhesions that may complicate to re-do surgeries. Embolization, and viral-transmission have been rarely reported^[3,5]^.

OPCAB is the most common surgical method of coronary revascularization in the developing world. This is a rare complication associated with OPCAB. Surgeons practicing OPCAB should be judicious in patient selection and should avoid operating on patients with recent myocardial ischemia rendering the friable myocardium.

## CONCLUSION

It is good to perform safe surgeries, however, even better to be watchful about the unusual complications and be prepared to manage them.

In desperate times, cyanoacrylate may prove to be successful in controlling an otherwise unmanageable situation.

**Table:** 

Author's roles & responsibilities	
AKP	Substantial contributions to the conception or design of the work; or the acquisition, analysis, or interpretation of data for the work; drafting the work or revising it critically for important intellectual content; final approval of the version to be published
MG	Substantial contributions to the conception or design of the work; or the acquisition, analysis, or interpretation of data for the work; drafting the work or revising it critically for important intellectual content; final approval of the version to be published
AKS	Substantial contributions to the conception or design of the work; or the acquisition, analysis, or interpretation of data for the work; drafting the work or revising it critically for important intellectual content; final approval of the version to be published
KKS	Substantial contributions to the conception or design of the work; or the acquisition, analysis, or interpretation of data for the work; drafting the work or revising it critically for important intellectual content; final approval of the version to be published
